# Abortion-related morbidity in six Latin American and Caribbean countries: findings of the WHO/HRP multi-country survey on abortion (MCS-A)

**DOI:** 10.1136/bmjgh-2021-005618

**Published:** 2021-08-20

**Authors:** Mariana Romero, Rodolfo Gomez Ponce de Leon, Luiz Francisco Baccaro, Berenise Carroli, Hedieh Mehrtash, Jimena Randolino, Elisa Menjivar, Erika Estevez Saint-Hilaire, Maria del Pilar Huatuco, Rosalinda Hernandez Muñoz, Gabriela Garcia Camacho, Soe Soe Thwin, Liana Campodonico, Edgardo Abalos, Daniel Giordano, Hugo Gamerro, Caron Rahn Kim, Bela Ganatra, Metin Gülmezoglu, Özge Tuncalp, Guillermo Carroli

**Affiliations:** 1 Health, Economy and Society Department, CEDES, Buenos Aires, Argentina; 2 CONICET, Buenos Aires, Argentina; 3 Latin American Center for Perinatology/Women's Health and Reproductive Health, Pan American Health Organization (CLAP/WR-PAHO/WHO), Montevideo, Uruguay; 4 UNICAMP, Campinas, Brazil; 5 CREP, Rosario, Santa Fe, Argentina; 6 UNDP/UNFPA/UNICEF/WHO/World Bank Special Programme of Research, Development and Research Training in Human Reproduction (HRP), Department of Sexual and Reproductive Health and Research, WHO, Geneva, Switzerland; 7 Pan American Health Organization El Salvador, San Salvador, El Salvador; 8 Hospital Materno Infantil San Lorenzo de los Mina, Santo Domingo, Dominican Republic; 9 ESSALUD, Lima, Peru; 10 Pan American Health Organization Bolivia, La Paz, Bolivia, Plurinational State of

**Keywords:** maternal health, public health

## Abstract

**Introduction:**

Abortion-related complications are a significant cause of morbidity and mortality among women in many Latin American and Caribbean (LAC) countries. The objective of this study was to characterise abortion-related complication severity, describe the management of these complications and report women’s experiences with abortion care in selected countries of the Americas region.

**Methods:**

This is a cross-sectional study of 70 health facilities across six countries in the region. We collected data on women’s characteristics including socio-demographics, obstetric history, clinical information, management procedures and using Audio Computer-Assisted Self-Interviewing (ACASI) survey the experience of abortion care. Descriptive bivariate analysis was performed for women’s characteristics, management of complications and reported experiences of abortion care by severity of complications, organised in five hierarchical mutually exclusive categories based on indicators present at assessment. Generalised linear estimation models were used to assess the association between women’s characteristics and severity of complications.

**Results:**

We collected data on 7983 women with abortion-related complications. Complications were classified as mild (46.3%), moderate (49.5%), potentially life-threatening (3.1%), near-miss cases (1.1%) and deaths (0.2%). Being single, having a gestational age of ≥13 weeks and having expelled products of conception before arrival at the facility were significantly associated with experiencing severe maternal outcomes compared with mild complications.

Management of abortion-related complications included both uterotonics and uterine evacuation for two-thirds of the women while one-third received uterine evacuation only. Surgical uterine evacuation was performed in 93.2% (7437/7983) of women, being vacuum aspiration the most common one (5007/7437, 67.4%).

Of the 327 women who completed the ACASI survey, 16.5% reported having an induced abortion, 12.5% of the women stated that they were not given explanations regarding their care nor were able to ask questions during their examination and treatment with percentages increasing with the severity of morbidity.

**Conclusions:**

This is one of the first studies using a standardised methodology to measure severity of abortion-related complications and women’s experiences with abortion care in LAC. Results aim to inform policies and programmes addressing sexual and reproductive rights and health in the region.

**Key questions**

**What is already known?**

- Globally, Latin America and Caribbean (LAC) region has the most legally restrictive abortion laws and policies.
- Complications as a result of abortions account for 9.9% (8.1–13) of maternal deaths in the LAC region.
- Estimates of post-abortion complications indicate that the LAC has lower rates than Asia and Africa, with a regional rate of 5.3 per 1000 women aged 15–44 years (around 757 000 women per year) reflecting a decrease from previous estimates (7.7 in 2005).

**What are the new findings?**

- This is one of the few global studies to provide data on abortion-related complications, collecting data across 70 health facilities in six LAC countries using a standardised tool.
- This study provides insights on the burden and management of abortion-related complications in health facilities in LAC region, using a hierarchal severity gradient, according to sociodemographic, obstetric and clinical characteristics.
- Although most women in our study were classified as having mild or moderate complications, the proportion of women with potentially life-threatening complications and with severe maternal outcomes (including death) is still high.

**Key questions**

**What do the new findings imply?**

- The prevalence of these severe complications varies across countries illustrating that abortion continues to be a major public health and policy challenge to address in the LAC region.
- Future research in the region should focus on measurement of abortion-related complications using the standardised methodology proposed in this study to document the severity of abortion-related complications in the region.

## Introduction

Estimates suggest that between the years 2015 and 2019, around 73 million abortions occurred worldwide annually.^1^ Based on figures from 2014, almost a half of abortions were unsafe, with 97% taking place in developing countries.^2^ However, what constitutes abortion safety has been an evolving discussion, particularly after the evidence-based WHO recommendations related to methods, providers and settings, based on gestational age, was published.^3–5^ In the advent of misoprostol and mifepristone, availability of information and access to these medications for women, WHO has been developing and updating its guidelines on abortion and post-abortion care.^5–7^ Abortion safety has been recognised as a multidimensional concept that taps into the continuum of existing risks and takes into account social determinants such as the legal context, access and equity. As a result, a theoretical framework was developed where abortion safety is classified in three groups: safe, less safe and least safe.^8^ By applying these categories, Latin America has the highest proportion of ‘less safe’ abortion among all regions.^2^

This data become relevant in the light of an evolving scenario where women and providers have started to switch from unsafe methods to misoprostol, reducing the severity of complications due to its effectiveness and safety. Estimates of post-abortion complications indicate that the Latin American region has lower rates than Asia and Africa, with a regional rate of 5.3 per 1000 women aged 15–44 years (around 757 000 women per year) reflecting a decrease from previous estimates (7.7 in 2005).^4^ Rates of post-abortion complications per 1000 women aged 15–44 years range from 2.4 in Brazil to 10.3 in Dominican Republic.^4^ However, official data may not be reliable enough to inform programmatic and policy decisions.^9^ Estimations stem from national statistics on hospital discharges, with the majority being from the public health sector, which can lead to under-reporting due to the sensitivity of the issue and the difficulties in capturing the true nature of the reported abortions (spontaneous or induced) in the region.^3 10 11^

To better understand the context, it is important to highlight that the Latin America and Caribbean (LAC) region has the most legally restrictive abortion laws and policies globally, limiting the provision of safe abortion and post-abortion quality care. Across the region, abortion is legal on request in three countries only (Cuba, Uruguay and Guyana), while abortion is prohibited in all circumstances in four countries (El Salvador, Honduras, Nicaragua and the Dominican Republic).^12^ It has been shown that there is an association between proportion of unsafe abortion and highly restrictive laws, suggesting that an enabling environment and legal grounds play a role in abortion safety.^8^ The WHO *safe abortion: technical and policy guidance for health systems* recommends that regulatory, policy and programmatic barriers that hinder access to and timely provision of safe abortion care should be removed.^4^ Lately attempted changes in penal codes like in the case of Bolivia trying to legalise abortion up to 8 weeks or abortion legalisation in Argentina up to 14 weeks, that created a ‘green wave’ across the continent, are promoting change at the national and regional levels.^13^

At the same time, women’s receipt of person-centred and respectful abortion care has also become an area of growing of research interest in the region.^14^ However, as pointed out by Darney *et al*, very little evidence exists documenting client perceptions of both technical or interpersonal quality, especially from low- and middle-income country settings.^15^

In an effort to capture accurate information surrounding abortion-related complications and post-abortion care, WHO/HRP conducted the multi-country survey on abortion (MCS-A)-related morbidity to evaluate the burden and severity of abortion-related complications and management among women presenting to health facilities in countries from Africa and LAC. We also explored abortion safety characteristics according to WHO definition and the experience of care reported by women.^7^ This paper reports the results of this survey in six countries from the LAC region.

## Methods

### Study design and participants

The protocol of the WHO/HRP MCS-A study is published elsewhere.^16^ Briefly, it is a large cross-sectional study with prospective data collection across 280 health facilities from 17 countries in Africa and LAC regions. In this article, we focus on the findings from 70 health facilities across six LAC countries: Argentina, Bolivia, Brazil, Dominican Republic, El Salvador and Peru. Facilities were identified through a multistage sampling method once countries were selected. A second stage of sampling consisted of random selection of two provinces/states, with probability proportional to the population size, plus the capital city/metropolitan area. Once the geographical areas were selected, 10 facilities per state/province (up a total of 30 facilities per country) from the census of private and public facilities were selected.

Health facilities were eligible if they fulfilled the following characteristics: >1000 deliveries per year, gynaecology ward, surgical capability to provide emergency obstetrical care including removal of retained products and, abortion provision and/or post-abortion care based on the facility assessment forms (online supplemental annex I). To ensure sufficient data contribution, facilities reporting less than 10 post-abortion care patients per month were excluded.

10.1136/bmjgh-2021-005618.supp1 Supplementary data

A hospital administrator or a healthcare provider responsible for the gynaecology and obstetrics wards at each participating facility provided institutional-level data. All women presenting to the facility with signs and symptoms from abortion-related complications or early pregnancy loss (including ectopic and molar pregnancies) or death at discharge were included for medical record review. Women with a diagnosis of threatened abortion, defined as vaginal bleeding with a closed cervix were excluded. The criterion of reviewing medical records of all women presenting to the facilities, rather than admissions, was used to avoid exclusion of those seeking care for mild complications. Included women were eligible for the exit survey if admitted for a minimum of 24 hours and experienced at least one of the following conditions: infection, haemorrhage/anaemia, perforated organs or injury to reproductive organs, complications resulting in operative management, and were able and willing to consent.

We obtained written informed consent (WIC) from all women participating in the exit surveys. No WIC was requested to collect data from medical records except for Bolivia and Brazil where National Institutional Review Board indicated that WIC must be obtained from every woman accounted in the study. Institutional informed consent was obtained from the responsible authority of each facility. Research Ethical Review Committee at WHO and at health authorities of each country, as well as those of all hospitals, independently approved the protocol.

### Data collection and data management

Trained research assistants abstracted socio-demographic and clinical information from medical records including age, marital status, education, obstetrics characteristics, signs and symptoms of abortion-related complications, medical procedures, clinical outcomes and vital status at discharge. We transcribed data into paper-based case report forms and entered it into a web-based electronic data capture system developed by the Centro Rosarino de Estudios Perinatales. Data for the exit survey were collected using Audio Computer-Assisted Self-Interviewing (ACASI), a system developed by Tufts University.^17^ ACASI data consisted of abortion safety characteristics (method used, provider, setting), and experience of abortion care related to effective communication, respect and dignity and emotional support. As in previous WHO multi-country surveys, data managers in Argentina monitored the study data flow and validated data quality. In each country, data collection took place over a 3-month period between June 2018 and January 2019.

### Statistical analysis

**Figure 1:**
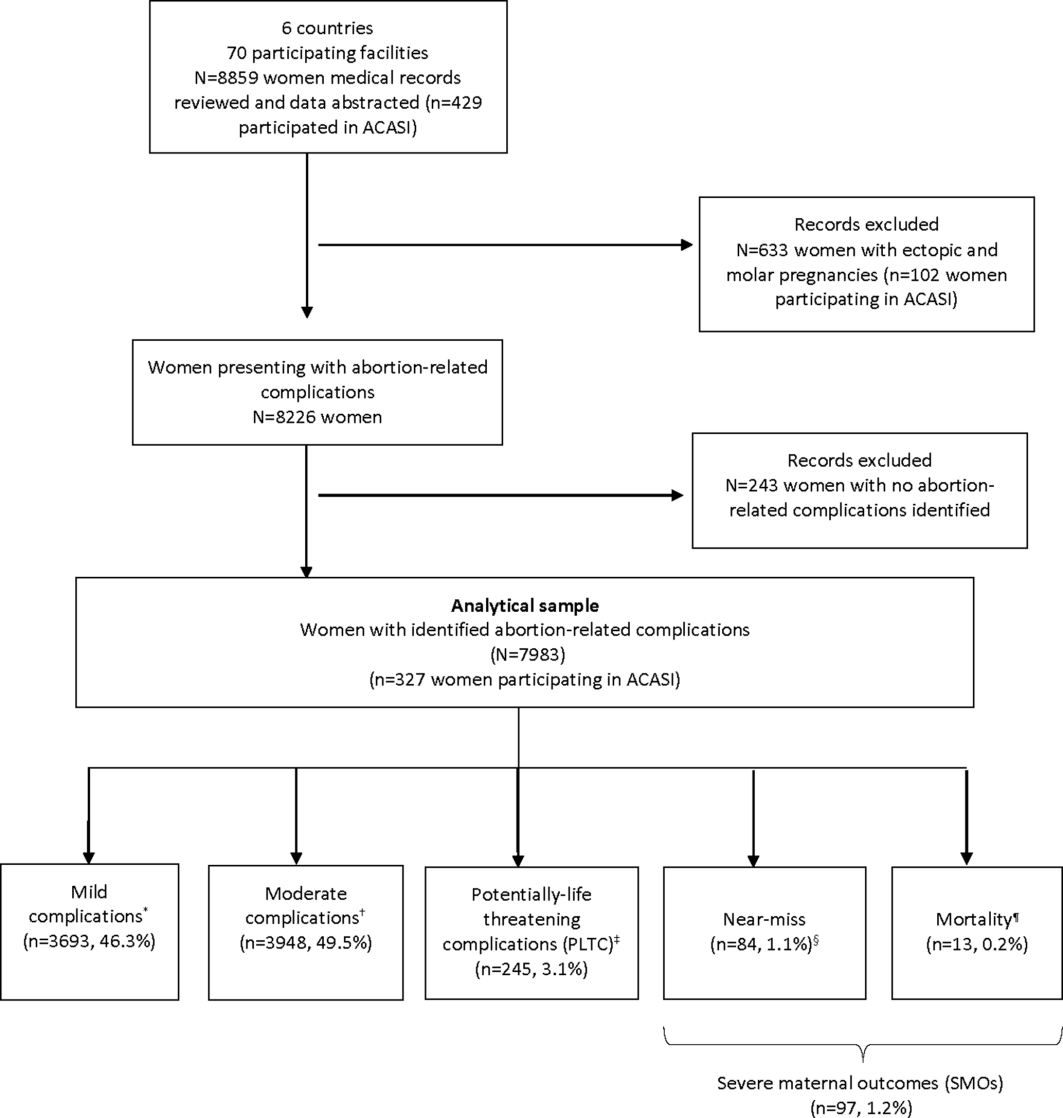
Study flow diagram for severity of abortion-related complications across six Latin American and Caribbean countries. ACASI, Audio Computer-Assisted Self-Interviewing. *Mild complications based on abnormal physical examination findings on initial assessment (vital signs, appearance, mental status, abdominal examination, gynaecological examination); †Moderate complications (heavy bleeding, suspected intra-abdominal injury, or infection); ‡WHO potentially-life threatening conditions (severe haemorrhage, severe systemic infection or suspected uterine perforation); §WHO maternal near-miss criteria (organ dysfunction of either one or more of the following: cardiovascular, respiratory, renal, coagulation, hepatic, neurologic or uterine dysfimction); ¶Status at discharge.

Based on clinical indicators at hospital admission, laboratory markers and management-based indicators during hospitalisation up to discharge or death, women were grouped into five hierarchical categories: mild complications, moderate complications, potentially-life threatening complications, near-miss and deaths (figure 1). Based on WHO identification criteria for maternal near-miss and potentially life-threatening complications (PLTC), we combined mortality and near-miss complications as severe maternal outcomes (SMO).^18^ Identification criteria are shown in online supplemental annex II. Furthermore, using the WHO Global Abortion Policies Database, we described the legal environments surrounding abortion provision, medication availability and abortion protocols across the six countries (online supplemental annex III).^12^

We performed descriptive analysis on national and facility level characteristics, as well as socio-demographic, obstetric and clinical management factors, by comparing proportions of characteristics across abortion-related complication severity categories using χ^2^ test. Country specific severity burden of abortion-related complications was computed as number of complications per 1000 women for each severity category and their 95% CIs estimated.

To determine women’s characteristics potentially associated with abortion-related complications, separate generalised linear regression models were fitted to estimate the odds of moderate, potentially life-threatening and severe complications compared with mild category for socio-demographic and obstetric characteristics (age, marital status, prior pregnancies, gestational age and expulsion of the products of conception (POC) before arrival at the facility), adjusting for potential clustering effect by country.

Gestational age at presentation was grouped as <13 weeks, ≥13 weeks or undetermined weeks. We categorised the clinical management of abortion-related complications as managed by uterotonics only, by uterine evacuation only, or by both methods. We further divided uterotonic use into single agents and their combination; and uterine evacuation by type of procedure: vacuum aspiration, dilation and curettage (D&C), or both.

For self-reported data collected by ACASI, we performed descriptive analysis to evaluate the abortion methods used, information received and help sought to end pregnancy. Experience of abortion care during facility stay was assessed by comparing responses across severity of abortion-related complications using χ^2^ test. Data analysis was conducted using SAS (V.9.4).

## Results

We collected data from 70 facilities in six LAC countries. Forty-one (57.8%) were tertiary level facilities. Further details of the health facilities characteristics can be found in online supplemental annex I.

A total of 8859 women seeking care with signs and symptoms of abortion-related complications or early pregnancy loss (including ectopic and molar pregnancies) were included, from which 8226 (92.9%) had complications of abortions and 633 (7.1%) had complications of molar or ectopic pregnancies. This analysis focused on abortion-related complications, therefore we excluded molar and ectopic pregnancies. We further excluded 243 cases because severity could not be determined. The final analysis included data on 7983 women with abortion-related complications (figure 1).

Based on the inclusion criteria, 484 women with abortion-related complications were eligible for ACASI, from which 327 (67.5%) consented to participate in the exit interview. Of note, all eligible women from El Salvador declined to participate. Eligibility criteria and participation per country can be found in online supplemental annex IV.

### Severity of abortion-related complications

**Figure 2:**
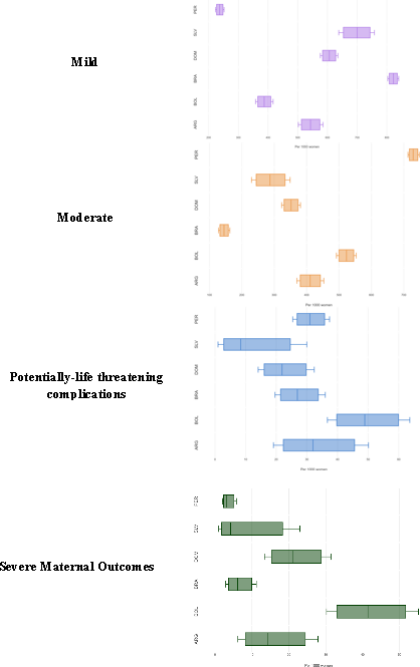
Severity of abortion-related complications by country. ^1^Figures are drawn to scale for each severity category. ARG, argentina; BOL, bolivia; BRA, brazil; DOM, dominican republic; SLV, El Salvador; PER, peru.

From 7983 women who had abortion-related complications, 46.3% had mild complications, 49.5% had moderate complications, 3.1% had PLTC, 1.1% of women were identified as near-miss cases and there were 13 deaths (0.2%) (figure 1). Deaths and near-miss were grouped as SMO, totalling 97 cases (1.2%). Across countries, severity burden for SMO and PLTC were higher for Bolivia while moderate and mild complications were higher for Peru and Brazil, respectively (figure 2).

Most women seeking care for abortion complications were seen in facilities from countries where abortion is permitted only under certain indications. Some of these countries have National Guidelines for both Induced Abortion and Post-Abortion Care in use (table 1). Misoprostol is approved and registered for gynaecological indications except in El Salvador where it is not registered but included in the National List of Essential Medicines. Mifepristone is not registered in any of the study countries. Approximately 96% of women were treated at secondary or tertiary level facilities, predominantly in an urban setting.

**Table 1:** Legal status of abortion among six participating Latin American and Caribbean countries

Legal classification	Country
Law prohibits all abortion	Dominican Republic, El Salvador
Unlawful abortion is prohibited or where there are only penalties for unlawful abortion, with no additional information provided about lawful abortion	None
Law allows or permits abortion only on one or more legal grounds	Argentina, Bolivia, Brazil, Peru
Law entitles a woman to abortion on request with no requirement for justification	None

Women presenting to facilities with abortion-related complications were predominantly between 20 and 29 years old, married or cohabitating and reporting secondary education or above. Only one-third of them were employed gainfully. Distribution of these sociodemographic factors were observed to be significantly different across the severity groups (table 2).

**Table 2:** National, sociodemographic and obstetrical characteristics of study population by severity of abortion-related complications

	Total (N=7983)	Mild (N=3693)	Moderate (N=3948)	Potentially life-threatening complications (N=245)	Severe maternal outcomes (N=97)
**National**					
Misoprostol country recognised approval†**					
No	237 (2.9)	166 (4.5)	68 (1.7)	2 (0.8)	1 (1)
Yes	7746 (97)	3527 (95.5)	3880 (98.3)	243 (99.2)	96 (98.9)
National guidelines on abortions‡**					
Post-abortion care (PAC) Only	1332 (16.7)	829 (22.5)	453 (11.5)	26 (10.6)	24 (24.7)
Both (induced abortion and PAC)	6651 (83.3)	2864 (77.6)	3495 (88.5)	219 (89.4)	73 (75.3)
**Facility**					
Facility type**					
Primary	288 (4.3)	269 (7.4)	19 (0.7)	0	0
Secondary	2238 (33.7)	1086 (29.9)	1024 (37.9)	99 (43.4)	29 (30.9)
Tertiary	4120 (61.9)	2267 (62.6)	1659 (61.4)	129 (56.6)	65 (69.2)
Other referral level	1337 (16.8)	71 (1.9)	1246 (31.6)	17 (6.9)	3 (3.1)
Location**					
Urban	7252 (90.9)	3224 (87.3)	3698 (93.7)	235 (95.9)	95 (97.9)
Peri-urban	675 (8.5)	438 (11.9)	225 (5.7)	10 (4.1)	2 (2.1)
Rural	56 (0.7)	31 (0.8)	25 (0.6)	0	0
**Sociodemographic and obstetrical**					
Age (in years)*	7963				
≤19	964 (12.1)	500 (13.6)	429 (10.9)	24 (9.8)	11 (11.5)
20–29	3720 (46.7)	1705 (46.3)	1850 (46.9)	118 (48.4)	47 (48.9)
≥30	3279 (41.2)	1476 (40.1)	1663 (42.2)	102 (41.8)	38 (39.6)
Marital status*	7356				
Single	2355 (32)	1146 (48.7)	1099 (46.7)	78 (34.9)	32 (34.8)
Married/cohabitating	4871 (66.1)	2211 (64.2)	2452 (68.2)	140 (62.8)	58 (63)
Separated/divorced/widowed	140 (1.9)	86 (2.5)	47 (1.3)	5 (2.2)	2 (2.2)
Education*	6736				
No education	72 (1.1)	42 (1.3)	26 (0.8)	1 (0.5)	3 (3.8)
Primary	865 (12.8)	514 (16.3)	308 (9.4)	25 (12.4)	18 (22.8)
Secondary or more	5799 (86.1)	2605 (82.4)	2960 (89.9)	176 (87.1)	58 (73.4)
Gainful occupation*	6441				
Yes	2017 (31.3)	987 (31.8)	926 (30.2)	73 (39.3)	31 (38.3)
No	4424 (68.7)	2116 (68.2)	2145 (69.9)	113 (60.8)	50 (61.7)
Previous pregnancies*	7944				
1 or more	5960 (75)	2771 (75.6)	2912 (74)	199 (81.2)	78 (80.4)
0	1984 (24.9)	896 (24.4)	1023 (26)	46 (18.8)	19 (19.6)
Previous abortions (in women reporting previous pregnancies)	5958				
1 or more	2451 (41.1)	1169 (42.2)	1169 (40.1)	76 (38.4)	37 (47.4)
0	3507 (58.9)	1601 (57.8)	1743 (59.9)	122 (61.6)	41 (52.3)
Gestational age (in weeks)**					
<13	5886 (73.7)	2808 (76)	2856 (72.3)	159 (64.9)	63 (64.9)
≥13	995 (12.5)	515 (13.9)	405 (10.3)	56 (22.9)	19 (19.6)
Undetermined	1102 (13.8)	370 (10)	687 (17.4)	30 (12.2)	15 (15.5)
Expulsion of products of conception before arrival to facility**	7716				
Yes	3061 (39.7)	1284 (35.7)	1579 (41.6)	144 (62.6)	54 (58.7)
No	4655 (60.3)	2313 (64.3)	2218 (58.4)	86 (37.4)	38 (41.3)

*P value<0.05; **p value<0.0001.

†None: El Salvador (although misoprostol is listed in the National List of Essential Medicines) / misoprostol: Argentina, Bolivia, Brazil, Dominican Republic, Peru / mifepristone: not registered in any of the countries.

‡PAC only: Dominican Republic, El Salvador / Both: Argentina, Bolivia, Brazil, Peru.

Moreover, three out of four women had a previous pregnancy and approximately two in five women (41.1%) reported having had a prior abortion. Additionally, three-quarters of women presenting with abortion-related complications were determined to be at <13 weeks gestational age and significantly greater proportion of women in the SMO or potentially life-threatening groups had expelled products of conception prior to arrival at the facility compared with those in the mild or moderate groups (58.7% and 62.6% vs 41.6% and 35.7%, respectively).

Table 3 presents generalised linear models evaluating factors potentially associated with increased risk of severe abortion-related complications.^19 20^ Being single (adjusted OR=1.9; 95% CI 1.1 to 3.3), presenting with gestational age ≥13 weeks (adjusted OR=1.8; 95% CI 1.2 to 2.7) and having expulsion of products of conception before arrival at the facility (adjusted OR=1.8; 95% IC 1.3 to 2.4) were significantly associated with experiencing SMOs compared with mild complications, after adjusting for country clustering effect, and potential confounding by age, marital status, prior pregnancy, gestational age and expulsion of products of conception. Comparing women with PLTC to those with mild complications, expulsion of POC before arrival to the facility was found to be the single factor associated with increased severity of PLTC (adjusted OR=3.2; 95% CI 2.4 to 4.7), after adjusting for country clustering effect, age, marital status, prior pregnancy and gestational age. There were no differences between moderate and mild complications.

**Table 3:** Determinants* of increased risk of abortion-related complication severity compared with mild complications

		Moderate† versus mild‡		PLTC§ versus mild‡		SMO¶ versus mild‡	
		AOR	95% CI	AOR	95% CI	AOR	95% CI
Age (in years)	≤19 years	0.8	0.7 to 1	0.9	0.7 to 1.1	0.9	0.5 to 1.8
	20–29 years	0.9	0.9 to 1	1	0.7 to 1.6	0.9	0.6 to 1.3
	≥30 years	Reference	Reference	Reference	Reference	Reference	Reference
Marital status	Single	1.2	0.9 to 1.8	1.5	0.9 to 2.7	1.9**	**1.1 to 3.3**
	Other than single	Reference	Reference	Reference	Reference	Reference	Reference
Prior pregnancies	1 or more	0.9	0.8. to 0.9	1.3	0.9 to 1.9	1.3	0.8 to 1.9
	0	Reference	Reference	Reference	Reference	Reference	Reference
Gestational age (in weeks)	≥13	0.7	0.4 to 1.5	2.2	0.8 to 6.3	1.8**	**1.2 to 2.7**
	Undetermined	0.7	0.5 to 1.1	0.9	0.3 to 2.7	0.79	0.6 to 1.5
	<13	Reference	Reference	Reference	Reference	Reference	Reference
Expulsion of products of conception before arrival to facility	Yes	1.2	0.7 to 2.2	3.2**	**2.2 to 4.7**	1.8**	**1.3 to 2.4**
	No	Reference	Reference	Reference	Reference	Reference	Reference

*Models clustered around country.

†Moderate complications (heavy bleeding, suspected intra-abdominal injury or infection).

‡Mild complications (based on abnormal physical examination findings on initial assessment (vital signs, appearance, mental status, abdominal examination, gynaecological examination)).

§PLTC, potentially-life threatening complications (WHO potentially-life threatening conditions (severe haemorrhage, severe systemic infection or suspected uterine perforation)).

¶SMOs, severe maternal outcomes (WHO near-miss criteria and mortality).

**P value <0.05.

AOR, adjusted OR.

### Management of abortion-related complications

Table 4 details the management of abortion-related complications by severity group. Overall, almost two-third of women received both uterotonics and uterine evacuation, one-third received uterine evacuation only and very few (3.9%) received uterotonics only. In the group of women with SMO the use of uterotonics only was higher, and the use of uterine evacuation only was lower than in the rest of morbidity categories.

**Table 4:** Uterotonics and uterine evacuation for management of abortion-related complications by severity†

Management of complications	Total (N=7983)	Severity of abortion-related complications				
		Mild (N=3693)	Moderate (N=3948)	Potentially life-threatening complications (N=245)	Severe maternal outcomes (N=97)	P value
Uterotonics	315 (3.9)	183 (4.9)	116 (2.9)	8 (3.3)	8 (8.3)	<0.0001
Uterine evacuation	2565 (32.1)	1161 (31.4)	1299 (32.9)	82 (33.5)	23 (23.7)	
Both uterotonics and uterine evacuation	4872 (61)	2206 (59.7)	2460 (62.4)	148 (60.4)	58 (59.8)	
Other	90 (1.1)	47 (1.3)	31 (0.8)	5 (2)	7 (7.2)	
None	141 (1.8)	96 (2.6)	42 (1.1)	2 (0.8)	1 (1)	

*P value <0.0001 based on χ^2^ for each management category by severity.

†Mutually exclusive.

Most women seeking care for abortion complications received uterine evacuation (7437/7983, 93.2%) with D&C performed on approximately one-third (2360/7437, 31%). Among women with PLTC, the combination of vacuum aspiration and D&C was substantially higher (5.2%) than in mild, moderate and severe health outcomes groups (0.36%, 0.45% and 1.2%, respectively).

Only 3.3% of women received blood transfusion, and less than 1% needed a surgical intervention. Massive transfusions (>3 units) and surgeries were more frequent among severe cases, as well as the admission to intensive care unit (ICU) (table 5).

**Table 5:** Types of management by severity of abortion-related complications†

	Total (N=7983)	Severity of abortion-related complications			
		Mild (N=3693)	Moderate (N=3948)	Potentially life-threatening complications (N=245)	Severe maternal outcomes (N=97)
Uterotonics*	5187 (64.9)	2389 (64.7)	2576 (65.3)	156 (63.7)	66 (68)
Misoprostol	2207 (42.6)	709 (29.7)	1470 (57.1)	20 (12.8)	8 (12.1)
Oxytocin	801 (15.4)	366 (15.3)	364 (14.1)	56 (35.9)	15 (22.7)
Oxytocin and ergometrine	782 (15.1)	328 (13.7)	402 (15.6)	34 (21.8)	18 (27.3)
Ergometrine	712 (13.7)	536 (22.4)	145 (5.6)	20 (12.8)	11 (16.7)
Misoprostol and oxytocin	340 (6.6)	217 (9.1)	99 (3.8)	99 (3.8)	217 (9.1)
Misoprostol and ergometrine	194 (3.7)	156 (6.5)	35 (1.4)	1 (0.6)	2 (3)
Misoprostol, oxytocin and ergometrine	135 (2.6)	69 (2.9)	56 (2.2)	6 (3.9)	4 (6.1)
Other	16 (0.31)	8 (0.33)	5 (0.19)	2 (1.3)	1 (1.3)
Uterine evacuation*	7437 (93.2)	3367 (91.2)	3759 (95.2)	230 (93.9)	81 (83.5)
Vacuum aspiration	5009 (67.4)	2010 (59.7)	2818 (74.9)	127 (55.2)	54 (66.7)
Dilation and curettage (D&C)	2360 (31.7)	1330 (39.5)	913 (24.3)	91 (39.6)	26 (32.1)
Both vacuum aspiration and D&C	42 (0.56)	12 (0.36)	17 (0.45)	12 (5.2)	1 (1.2)
Other	26 (0.35)	15 (0.45)	11 (0.29)	0	0
Blood transfusion*	261 (3.3)	32 (0.87)	104 (2.6)	99 (40.4)	26 (26.8)
1 unit	83 (31.8)	10 (31.3)	41 (39.4)	28 (28.3)	4 (15.4)
2 units	125 (47.9)	16 (50)	52 (50)	47 (47.5)	10 (28.5)
3 units or more	53 (20.3)	6 (18.8)	11 (10.6)	24 (24.2)	12 (46.2)
Surgical procedures*	38 (0.48)	13 (0.35)	12 (0.30)	5 (2)	8 (8.3)
Laparoscopy	4 (10.5)	1 (7.7)	2 (16.7)	0	1 (12.5)
Exploratory laparotomy	10 (26.3)	5 (38.5)	2 (16.7)	2 (40)	1 (12.5)
Hysterectomy	24 (63.2)	7 (53.9)	8 (66.7)	3 (60)	6 (75)
Antibiotics received for prophylaxis or treatment*	5299 (66.4)	2114 (57.2)	2906 (73.6)	207 (84.5)	72 (74.2)
Admission to intensive care unit*	33 (0.4)	8 (0.22)	5 (0.13)	6 (2.5)	14 (14.4)

*P value<0.0001.

†Not mutually exclusive.

**Figure 3:**
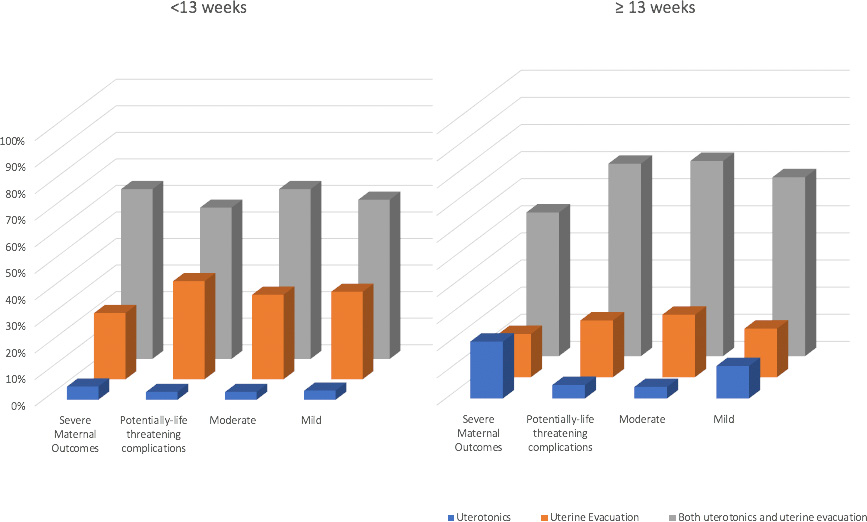
Use of uterotonics and uterine evacuation for management of abortion-related complications, by severity and gestational age.

Figure 3 describes the use of uterotonics and uterine evacuation for the management of abortion-related complications by gestational age (<13 and ≥13 weeks). Women were managed similarly across both gestational age groups, irrespective of their severity. There was a higher use of uterine evacuation only in earlier pregnancies (<13 weeks), and lower use of uterotonics alone in gestations ≥13 weeks, except in the cases of SMO.

### Women’s self-reported experiences of care via ACASI

Overall, 327 women participated in the ACASI exit interviews, and the distribution of sociodemographic and obstetrical characteristics of these women were not significantly different from the overall study population except for gainful occupation, gestational age and expulsion of POC before arrival (online supplemental annex V). A greater proportion of women who responded ACASI had ≥13 weeks of pregnancy (29.1% vs 12.5%) and had expelled products of conception before arrival (50.9% vs 39.7) which may reflect the need for a longer stay at the hospital as indicated in the eligibility criteria for ACASI.

Of 327 women, 54 (16.5%) reported an induced abortion. Among those, 46.3% stated that they did not receive information from anyone about methods used to end pregnancy. The most commonly reported sources of information were friends (29.6%), medical doctor (22.2%), someone else (18.5%) or husband/partner/boyfriend (16.7%). (table 6). In terms of seeking care from someone to end their pregnancy, 37% of the women reported not getting any help. The most reported assistance was from medical doctors (37%), a friend (24.1%) or someone else (16.7%) (online supplemental annex VI).

**Table 6:** Self-reported experience of abortion care during facility stay

	Total (N=327)	Severity of abortion-related complications			
		Mild (N=61)	Moderate (N=166)	Potentially life-threatening complications (N=87)	Severe maternal outcomes (N=13)
		N (%)	N (%)	N (%)	N (%)
Explanations regarding care and treatment (N=313)					
No	39 (12.5)	7 (11.7)	20 (12.6)	12 (14.8)	0
Able to ask questions during the examination and treatment (N=312)					
No	40 (12.8)	6 (10)	21 (13.3)	12 (14.8)	1 (7.7)
Feel doctor provided everything needed to know about decisions taken for care (N=310)					
No	48 (15.5)	8 (13.6)	27 (17.2)	12 (14.8)	1 (7.7)
Encountered anxiety or stress during hospital stay (N=312)					
Yes	239 (76.6)	48 (80)	120 (75.9)	61 (75.3)	10 (76.9)
If yes to above (N=239), not able to tell doctor or nurse who helped you that you were feeling anxious or stress	113 (47.5)	23 (47.9)	62 (52.1)	24 (39.3)	4 (40)
If yes to above (N=125), not offered additional support when you told the doctor or nurse about feeling the anxiety or stress	28 (22.8)	8 (32)	12 (21.4)	8 (22.2)	0
Feel choices and preferences were followed during hospital stay (N=312)					
No	44 (14.1)	6 (10)	23 (14.6)	15 (18.5)	0
Spoken to nicely (N=311)					
No	28 (9)	5 (8.3)	15 (9.6)	8 (9.9)	0
Receive pain medications during hospital stay (N=312)					
No	25 (8)	4 (6.7)	16 (10.1)	5 (6.2)	0
If yes (N=287), pain medications did not help ease pain	12 (4.2)	3 (5.4)	6 (4.2)	3 (3.9)	0

Table 6 presents experience of abortion care during facility stay as reported by women. Three out of four women encountered anxiety and stress during the hospital stay in similar proportions across severity. Overall, 12.5% of the women stated that they were not given explanations regarding their care and treatment and 12.8% women reported that they were not able to ask questions during their examination and treatment with percentages increasing with the severity of morbidity.

## Discussion

This paper reports the results of a study aimed at surveying abortion-related complications and its management in women seeking care to health facilities in six LAC countries. It also includes an assessment of the quality of care women received from their own perspective. The resulting regional sample included countries (Argentina, Bolivia, Brazil, Dominican Republic, El Salvador, Peru) with diverse scenarios in terms of the legal restrictions, availability of abortion medications and guidelines. Although most women in our study were classified as having mild or moderate complications, the proportion of women with PLTCs and with SMOs (including deaths) are still high. The prevalence of moderate, PLTC and SMO account for 53.8% illustrating that complications as a result of abortions continue to be a major public health and policy challenge to address in the LAC region.

The main findings from this study also revealed that severe abortion-related complications were associated with being single, later gestational age (≥13 weeks) and expulsion of POC prior to arrival to the facility. Although these findings show an association between each individual factor and morbidity outcomes, further analysis is required to understand the potential conditions and sequence of events leading to different results in the spectrum of severity. Several studies in the region concur with findings that support the association of being single or having a lower educational status with delays in accessing abortion care.^21 22^ Others show that even when misoprostol is available contributing to decrease abortion-related complications, barriers on accessing information on use, dosage and expected outcomes make women misinterpret warning signs affecting when to seek care.^23–25^

Even though the participating numbers were small, women’s responses in the ACASI exit survey showed that 46% did not have any previous information on how to end a pregnancy.^26 27^ Among those who stated having some information, friends and physicians were the most frequently reported source. Moreover, physicians or medical doctors were the most cited as having helped to end the pregnancy. This shows willingness of health professionals to provide help and information in restrictive contexts, which constitutes a rather new scenario probably due to regional initiatives that appealed to professionalism and ethics in relation to the duty of providing information as a way of reducing unsafe abortion practices.^28 29^

Our study is part of the larger WHO/HRP MCS-A that includes 11 countries in Africa using the same methodology allowing comparability.^30^ SMOs and PLTC reported for Africa (7%) are almost double those of our region (4.3%). Additionally, the majority of women in LAC were seeking post-abortion care at an earlier gestational age (under 13 weeks) than in Africa (73.7% vs 52.8%). These results support the findings related to abortion safety where in LAC most women seek care for unsafe abortions that could be categorised as less safe, whereas in Africa, almost all unsafe abortions were classified as least safe.^2^ Factors that might explain these differences in LAC could be the transition from the use of dangerous methods to misoprostol outside the formal health system, as well as a better access to care and treatment of abortion-related complications when they occur.^2 4 31^ Studies on the women’s perspectives show greater access to information and methods, and accompaniment, while on the providers’ side, less stigma and a high level of awareness of potential complications are seen.^32 33^ Although this transition has improved overall indicators, it is not homogeneous, nor stable in the region, and facilities are far from implementing WHO recommendations consistently in practice.^4 6^ Similar to the Africa region, D&C is still used in the management of abortion-related complications in LAC. Overall, 3 in 10 women received it despite longstanding efforts from WHO, PAHO/CLAP, FIGO and other agencies, to promote the use of safer uterine evacuation methods such as manual vacuum aspiration.^34^ The combined use of uterotonics and uterine evacuation for 6 out of 10 women seems to be high, and suggest probable over-medicalisation or overtreatment in all severity and gestational age groups. However, we have not explored individual medical indications nor justifications for combined or sequential medical/surgical treatments to confirm this. Nevertheless, the most frequently used uterotonic in LAC was misoprostol (42.6%), in contrast with the use of oxytocin by half of the cases reported in the Africa study.

Through the exit surveys, there is a similarity among women participating in both LAC and Africa MCS-A studies, regarding their perceptions as to how well they were informed about care and treatment by providers (87.5% and 79.9%) and feeling doctors provided everything needed to know about decisions taken for care (84.5% and 79.3%).^30^ Results are similar to those obtained in studies in Brazil, Argentina and Mexico that show an improvement in the proportion of women receiving information compared with previous studies.^35–37^ However, information sources differed between the two regions where information stemmed from friends and physicians in LAC whereas the internet and social media were highlighted as key sources in the Africa study. More than 7 out of 10 women participating in the study experienced post-abortion care with a degree of anxiety and stress. Fear of being mistreated, stigmatised or being reported, still persist in the region as revealed by several studies analysed by López Gómez, showing that the transition to a women centred care is still to be realised.^32^

To our knowledge, this is one of the few studies evaluating abortion morbidity and its management in the LAC region. Given the legal and political context, the available studies in the region address mostly maternal morbidity due to pregnancy and delivery using a cut point of 22 weeks gestational age and higher as eligibility criteria.^38 39^ The limited evidence from the region hinders the comparability of our findings. Standardised measurement of these complications in the LAC region is key to understand the extent of the burden that unsafe abortion poses on maternal morbidity and mortality reduction. In 2015, the Latin American Center for Perinatology and Women’s Reproductive Health (CLAP), a specialised centre from the PAHO, created a regional network of institutions in 16 countries, committed to improving healthcare and epidemiological surveillance for women receiving abortion care or facing a near-miss event using the Perinatal Information System (SIP). This network plays a very important role in applying the WHO recommendations in different legal settings and has reported results of post-abortion contraception and spontaneous abortion care.^40–42^

The restrictive legal and policy environment may serve as the determinant of abortion safety.^2^ Research has shown that globally, women have limited awareness and knowledge of the abortion laws and policy environment, even in countries with liberal laws, impeding women from accessing available services.^43^ In restrictive settings, such as the LAC region, abortion laws are sometimes worded in vague terms as to what is actually allowed in practice.^44^ The lack of transparency on abortion laws and policies and, the stigma surrounding abortion may lead women to seek delayed care or to avoid the health system entirely due to the lack of information on how and when to seek post-abortion care. The *WHO safe abortion: technical and policy guidance for health systems* recommends that regulatory, policy and programmatic barriers that hinder access to and timely provision of safe abortion care for all women should be removed.^44 45^ While access to safe and comprehensive abortion care, including post-abortion care is key, access to legal information is also critical. Future research using the information available on the *WHO Global Abortion Policies Database* may help to increase transparency and, to improve knowledge of providers and women’s understanding of abortion laws and policies to seek safe post-abortion care.^44^

### Strengths and limitations

This study reached a sample size of almost 8000 women presenting at public hospitals with abortion-related morbidity from six countries of LAC. It is the first study in the region to address this public health problem with a standardised classification based on WHO criteria. It not only collected data from clinical records but also interviewed women to assess their perceptions on quality of care. Another strength of the study is the inclusion of countries with varying degrees of restrictive abortion laws, in particular two countries (El Salvador and Dominican Republic) where abortion is penalised under all circumstances.

A limitation is that the contribution of each country to the sample may not reflect the true proportionality of the population, the country heterogeneity and the heterogeneity of institutions. For example, the most populated province of Argentina, Buenos Aires, refused to participate and it is worth noting that none of the women in El Salvador accepted to participate in ACASI. Pooling data across countries has enabled us to have sufficient data to look at some of the associations, but at the same time it may limit some of the analyses masking between-country differences.

Even though the severity of abortion-related complications among women who presented to the facilities varied across study countries, this being a cross-sectional profile may not be representative of the entire population, not only because many women may choose to remain at home, but also because the sample excluded private institutions, rural areas, and adolescents.

## Conclusions

This is one of the first studies of its kind and scope using a standardised methodology to measure severity of abortion-related complications and women’s experiences in their own voices in LAC and, will provide evidence to improve public policies addressing sexual and reproductive rights and health in the region. While showing a transition towards improved overall morbidity, results emphasise other areas of concern: persistence of curettage, limited use of misoprostol and limited awareness of women’s needs along the process of care. Future research in the region should focus on measurement of abortion-related complications using the standardised methodology developed in this study to document the severity of abortion-related complications. By measuring these complications and better understanding women’s reported experiences, we will be able to determine the extent of the burden of maternal morbidity and mortality and monitor the results of implementing public policies fostering universal health coverage and Sustainable Development Goals in the region.

## Data Availability

Data are available upon request. The data used for this analysis might be made available upon reasonable request, in accordance with the WHO/HRP MCS-A Research Group data sharing policy and WHO Policy of Data Use and Data Sharing. For further information, contact srhmph@who.int and srhpua@who.int.
