# Supplementary material for: Abortion-related morbidity in six Latin American and Caribbean countries: findings of the WHO/HRP multi-country survey on abortion (MCS-A)
Source: BMJ Glob Health. 2021 Aug 20;6(8):e005618. doi: 10.1136/bmjgh-2021-005618 (PMC8404437; doi:10.1136/bmjgh-2021-005618)
Supplement: Supplementary data [file bmjgh-2021-005618supp001.pdf]

MCSA LAC Main Paper

Supplementary Information

Annex I. Facility Characteristics

	Argentina	Bolivia	Brazil	Dominican Republic	El Salvador	Peru
**Number of participating facilities**	**13**	**11**	**20**	**7**	**5**	**14**
**Location**						
Urban	13	11	19	4	5	14
Rural	0	0	1	3	0	0
**Facility level**						
Primary	0	0	0	0	0	1
Secondary	4	7	8	1	4	3
Tertiary	8	4	11	6	1	9
Other	0	0	1	0	0	1
**Resources currently used at facility**						
Safe Abortion Guidance/Clinical Handbook	13 (100)	8 (72.7)	11 (61.1)	6 (85.7)	5 (100)	8 (57.1)
WHO guidelines (e.g. for postpartum haemorrhage)	13 (100)	8 (72.7)	17 (94.4)	5 (71.4)	5 (100)	10 (71.4)
Evidence-based, locally adapted guidelines	13 (100)	8 (72.7)	12 (66.7)	5 (71.4)	5 (100)	13 (92.9)
Clinical audits (e.g. maternal deaths reviews)	12 (92.3)	10 (90.9)	17 (94.4)	6 (85.7)	5 (100)	14 (100)
**Surgical abortion method employed for gestational age up to 12-14 weeks**	12 (92.3)	9 (81.8)	18 (90.0)	1 (14.3)	5 (100)	14 (100)
**Medical abortion offered at the facility (including medical management of incomplete abortion)[1]**	13 (100)	6 (54.6)	19 (95.0)	4 (57.1)	3 (60.0)	11 (78.6)
**Abortion for gestational age >13 weeks offered at facility**	12 (92.3)	7 (63.6)	16 (80.0)	5 (71.4)	1 (20.0)	10 (71.4)
**Post-abortion contraception offered at facility**	13 (100)	10 (90.9)	10 (55.6)	6 (85.7)	5 (100)	14 (100)

[1] Country recognized approval of misoprostol and mifepristone: None: El Salvador (although misoprostol is listed in the National Essential Medicines List) / Misoprostol: Argentina, Bolivia, Brazil, Dominican Republic, Peru / Mifepristone: not registered in any of the countries.

Romero M, *et al. BMJ Global Health* 2021; 6:e005618. doi: 10.1136/bmjgh-2021-005618

MCSA LAC Main Paper

Annex II. Criteria for hierarchal classification of abortion-related complications

**Mild (3693, 46.3%)**		**Moderate (3948, 49.5%)**		**Potentially-life threatening complications (PLTC) (245, 3.1%)**		**WHO Near-Miss Criteria (84, 1.1%)**	
**Signs and symptoms on initial assessment**	**N (%)**	**Symptoms**	**N (%)**	**Symptoms**	**N (%)**	**Organ dysfunction**	**N (%)**
Vaginal Bleeding	3056 (82.8)	Bleeding<br>*Heavy bright red vaginal bleeding (with or without clots), Blood soaked pads/towels/clothing, pallor*	3813 (96.6)	Severe haemorrhage	138 (56.3)	Cardiovascular	25 (29.8)
Cervix open	2238 (60.6)	Suspected intra-abdominal injury<br>*Abdominal pain/cramping, nausea, vomiting distended/tense/hard abdomen Shoulder pain Decreased bowel sounds, rebound, tenderness*	777 (19.7)	Severe systemic infection	115 (46.9)	Coagulation	23 (27.4)
Abnormal vital signs<br>*based on temperature, heart rate, systolic/diastolic blood pressure, and respiratory rate*	640 (17.3)	Infection<br>*Chills, fevers, sweats Foul smelling vagina discharge History of interference with pregnancy*	179 (4.5)	Uterine perforation	11 (4.5)	Neurologic	15 (17.9)
Uterine tenderness	463 (12.5)					Hepatic	15 (17.9)
Abnormal abdominal examination<br>*Rebounding/guarding. Distended, decreased bowel sounds, tense/hard, tenderness on palpitation*	485 (13.1)					Uterine	15 (17.9)
Cervical Motion Tenderness	356 (9.6)					Renal	12 (14.3)
Abnormal appearance<br>*Sick-looking, pallor, jaundice, clammy*	190 (5.1)					Respiratory	8 (9.5)
Abnormal mental status<br>*Agitated, lethargic, comatose*	187 (5.1)						
Foul smelling vaginal discharge	119 (3.2)						
Adnexal mass	12 (0.3)						
Evidence of foreign body	9 (0.2)						

Romero M, *et al. BMJ Global Health* 2021; 6:e005618. doi: 10.1136/bmjgh-2021-005618

Annex III. Development of variables within dataset

Abortion-related national, legal and policy environment
Considering the national, legal and policy environments for provision of safe abortion, a new open-access WHO Global Abortion Policies Database (GAPD) was launched [1]. It contains comprehensive information on the abortion laws, policies, health standards and guidelines for WHO and United Nations (UN) Member States, [1,2] . Using the open-access WHO GAPD, we described the national legal and policy environments surrounding abortion provision across the 6 Latin American and Caribbean countries in our study [2].

We report abortion-related complications by national level characteristics that included grouping countries according to 1) availability of national guidelines (none or post-abortion care only, or induced and post-abortion care guidelines) and, 2) country-recognized essential medicines for medical abortion (none or misoprostol only or misoprostol and mifepristone).

Timing of abortion-related complications based on facility admission
Presentation of abortion-related complication variables were based on clinical admission in the facility. The admission assessment response options were: yes at arrival or within 24 hours of facility stay or yes after 24 hours of facility stay. Less than 0.5% of responses indicated the presentation of the abortion-related complications occurred after 24 hours of facility stay. Therefore, clinical admission indicators for abortion-related complications are interpreted as at arrival/within 24 hours of facility stay for the purposes of the analyses.

Annex IV. ACASI Selection Criteria[1]

Women with abortion-related complications who are admitted for a minimum of 24 hours and able and willing to consent; plus experiencing at least one of the following conditions:

1) Infection (defined as foul smelling discharge, fever, peritonitis, required parenteral antibiotics for treatment)
2) Haemorrhage/anaemia (defined as estimated blood loss > 1000cc, haemoglobin blood transfusion, shock)
3) Perforated organs or injury to reproductive organs (i.e. uterine rupture, cervical lacerations)
4) Complications which result in operative management

Country	Women with ACASI criteria	Women answering ACASI	Response rate
Argentina	47	47	100%
Bolivia	77	44	57.1%
Brazil	109	70	64.2%
El Salvador	8	0	0%
Peru	191	112	58.6%
Dominican Rep.	52	52	100%
TOTAL	***484***	***327***	***67.5%***

[1]These numbers were calculated based on the inclusion criteria and available data on admission and discharge dates at the country level.

Annex V. National, sociodemographic and obstetric characteristics of ACASI study population by severity of abortion-related complications

	Entire population (N=7983)	ACASI (N=327)
**SOCIODEMOGRAPHIC AND OBSTETRIC**		
**Age (in years)**	**7963**	**327**
≤19	964 (12.1)	34 (10.4)
20 - 29	3720 (46.7)	160 (48.9)
≥30	3279 (41.2)	133 (40.7)
**Marital Status**	**7356**	**312**
Single	651 (21.9)	2852 (22.6)
Other than single	2315 (78.1)	9762 (77.4)
**Education**	**6736**	**293**
No education	72 (1.1)	4 (1.4)
Primary	865 (12.8)	49 (16.7)
Secondary or more	5799 (86.1)	240 (81.9)
**Gainful occupation**	**6441**	**271**
Yes	2017 (31.2)	108 (39.9)
**Previous pregnancies**	**7944**	**326**
0	1984 (24.9)	81 (24.9)
1 or more	5960 (75.0)	245 (75.2)
**Previous abortions**	**5958**	**245**
0	3507 (58.9)	142 (57.9)
1 or more	2451 (41.4)	103 (42.0)
**Gestational age (in weeks)***	**7983**	**327**
<13	5886 (73.7)	180 (55.1)
≥13	995 (12.5)	95 (29.1)
Undetermined	1102 (13.8)	52 (15.9)
**Expulsion of products of conception before arrival***	**7716**	**310**
Yes	3061 (39.7)	158 (50.9)

*p-value <0.0001; **p<0.05

Annex VI. Self-reported sources of information and help used to end pregnancy (N=54)

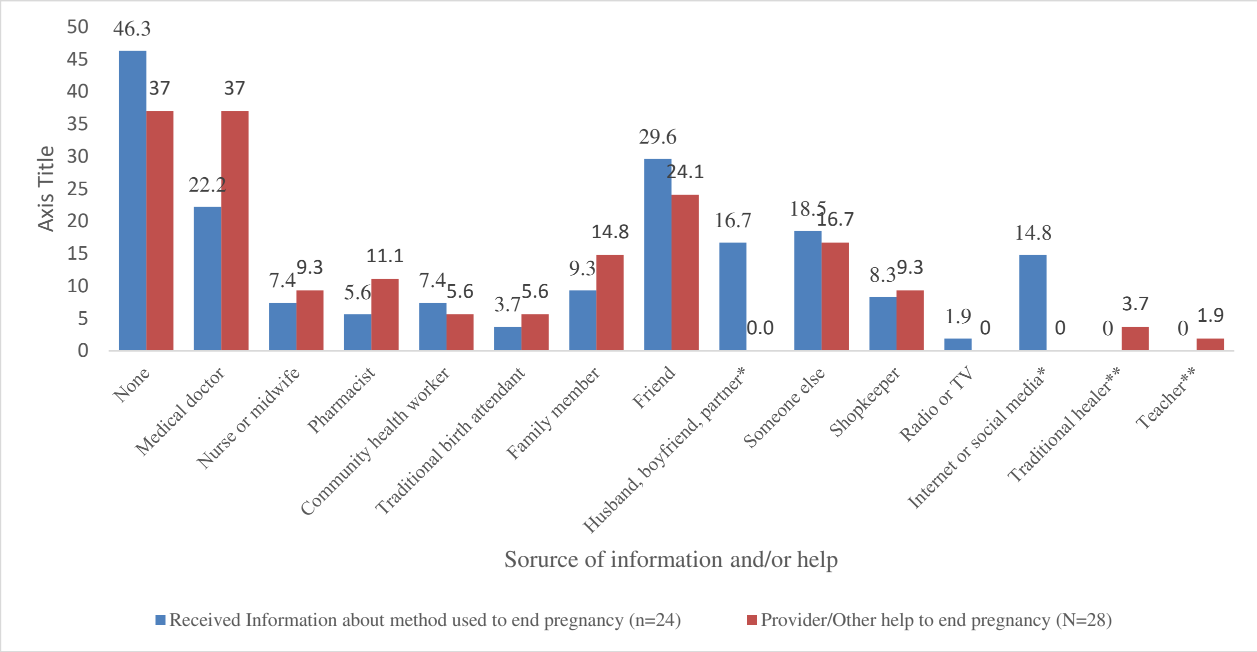

*Response option only included for information question
**Response option only included for help question

References

1. Johnson BR, Jr., Mishra V, Lavelanet AF, Khosla R, Ganatra B. A global database of abortion laws, policies, health standards and guidelines. *Bull World Health Organ* 2017; **95**(7): 542-4.
2. Johnson BR, Lavelanet AF, Schlitt S. Global Abortion Policies Database: a new approach to strengthening knowledge on laws, policies, and human rights standards. *BMC International Health and Human Rights* 2018; **18**(1): 35.
